# Selection of cereal-sourced lactic acid bacteria as candidate starters for the baking industry

**DOI:** 10.1371/journal.pone.0236190

**Published:** 2020-07-23

**Authors:** Vesna Milanović, Andrea Osimani, Cristiana Garofalo, Luca Belleggia, Antonietta Maoloni, Federica Cardinali, Massimo Mozzon, Roberta Foligni, Lucia Aquilanti, Francesca Clementi

**Affiliations:** Dipartimento di Scienze Agrarie, Alimentari ed Ambientali, Università Politecnica delle Marche, Ancona, Italy; Luleå University of Technology, SWEDEN

## Abstract

The quality of sourdough bread mainly depends on metabolic activities of lactic acid bacteria (LAB). The exopolysaccharides (EPS) produced by LAB affect positively the technological and nutritional properties of the bread, while phytases improve the bioavailability of the minerals by reducing its phytate content. In the present study, a pool of 152 cereal-sourced LAB were screened for production of phytases and EPS for potential use as sourdough starter cultures for the baking industry. There was large heterogeneity in the phytase activity observed among the screened isolates, with 95% showing the ability to degrade sodium phytate on plates containing Sourdough Simulation Medium (SSM). The isolates *Lactobacillus brevis* LD65 and *Lactobacillus plantarum* PB241 showed the highest enzymatic activity, while the isolates ascribed to *Weissella confusa* were characterized by low or no phytase activity. Only 18% of the screened LAB produced EPS, which were distinguished as ropy or mucoid phenotypes on SSM supplemented with sucrose. Almost all the EPS producers carried one or more genes (*eps*D/E and/or *eps*A) involved in the production of heteropolysaccharides (HePS), whereas the isolates ascribed to *Leuconostoc citreum* and *W*. *confusa* carried genes involved in the production of both HePS and homopolysaccharides (HoPS). Monosaccharide composition analysis of the EPS produced by a selected subset of isolates revealed that all the HePS included glucose, mannose, and galactose, though at different ratios. Furthermore, a few isolates ascribed to *L*. *citreum* and *W*. *confusa* and carrying the *gtf* gene produced β-glucans after fermentation in an *ad hoc* formulated barley flour medium. Based on the overall results collected, a subset of candidate sourdough starter cultures for the baking industry was selected, including *Lb*. *brevis* LD66 and *L*. *citreum* PB220, which showed high phytase activity and positive EPS production.

## Introduction

Cereal-based foods are an essential part of the daily diet worldwide, representing an important source of energy, carbohydrates and fiber, as well as proteins, minerals (e.g., zinc and magnesium) and micronutrients (e.g., vitamins B and E) to a lesser extent [1]. In western countries, the increasing consumer demand for cereal-based foods with improved nutritional and health properties has reshaped the wheat-to-bread sector. Sourdough undoubtedly represents a still valid option for the manufacturing of bread with improved texture, enhanced flavor, and prolonged shelf-life [2]. Sourdough is a mixture of flour and water spontaneously fermented or deliberately inoculated by lactic acid bacteria (LAB) and yeasts [3]. Sourdough starter cultures consisting of single or mixed strains of LAB and/or yeast with definite technological and/or functional traits are progressively being exploited by the high-tech baking industry [4–6].

LAB starter strains are primarily selected for the release of organic acids and carbon dioxide and for phytic acid degradation and/or exopolysaccharides (EPS) production [7]. Regarding this latter trait, numerous studies to date have noted the positive impact of *in situ*-produced EPS on both dough rheological parameters and bread qualities [8]. In a few of these studies, the EPS produced by LAB have been assayed as functional texturizers, showing a good potential to replace chemical additives or expensive ingredients (e.g., hydrocolloids) used as bread improvers [9, 10].

The functionality of the EPS is mainly affected by the composition, molecular weight, and structural conformation of these polymers [11]. In terms of composition, EPS can be classified as homopolysaccharides (HoPS) and heteropolysaccharides (HePS). HoPS are composed of only one type of monosaccharide, namely glucose or fructose, thereby being referred to as glucans (α or β) or fructans, respectively. β-glucans are of interest, as they are associated with health benefits [12]. Glucansucrases and fructansucrases, encoded by the glucansucrase (*gtf*) and levansucrase (*lev*) genes, respectively, catalyze the polymerization of glucans and fructans typically by using sucrose as the donor of the corresponding monosaccharide and transferring this residue to the reducing end of the growing HoPS [13]. HePS are composed of repeated units of at least two types of monosaccharides, mainly glucose, galactose, rhamnose, mannose, N-acetylglucosamine, N-acetyl galactosamine and rhamnose [14]. The biosynthesis of HePS is encoded by *eps* gene clusters, which encode the enzymes involved in the assemblage and transport of these polymers, as well as the regulation of such a process. A characteristic *eps* gene cluster is comprised of five highly conserved genes, *eps*A, *eps*B, *eps*C, *eps*D, and *eps*E, and a variable region including the genes for a polymerase (*wzy*), a flippase (*wzx*) and one or more glucosyltransferases and/or other polymer-modifying proteins [15].

While improving the textural qualities of bread, fermentation produced by LAB also results in improved bread mineral bioavailability and a reduced phytate content [16]. Phytic acid (myoinositol 1,2,3,4,5,6-hexakis dihydrogen phosphate) is the major storage form of phosphorus in cereal grains [17], where it concentrates in the bran fraction (corresponding to the aleurone layer and pericarp), thus explaining its higher abundance in whole and bran-enriched flours with respect to refined flours [18]. Phytic acid complexes with basic amino acids in proteins and acts as an excellent chelator of cations such as Ca^2+^, Mg^2+^, Fe^2+^ and Zn^2+^, which are made unavailable for absorption in the digestive system [19]. Phytic acid can be degraded by phytase (myo-inositol hexakisphosphate phosphohydrolase), an enzyme that catalyzes the gradual removal of orthophosphates from this compound. This enzyme is widely distributed in plants, animals and even microorganisms [20]. Flours produced from cereal grains contain endogenous phytases, but their levels are generally too low to lead to significant reductions in phytic acid levels [21]. It has been observed that acidification by sourdough LAB stimulates endogenous flour phytases that, together with phytase-producing LAB and yeasts, can significantly reduce the phytic acid content of bread [22]. For this reason, the exploitation of phytase-producing microorganisms in baking is very promising for the improvement of the bioavailability of minerals in cereal-based baked goods. Though several studies have explored the cloning and overexpression of phytase-encoding genes in lactobacilli [23], to the authors’ knowledge, no naturally occurring phytase genes have been monitored in these microorganisms, yet.

So far, numerous research papers and literature reviews have described the production of EPS by cereal-sourced LAB. However, only a few of these studies were aimed at analyzing the ability of these microorganisms to produce both phytases and EPS, with a focus on the molecular characterization of the produced EPS and the genes involved in their production. Moreover, there was a paucity of available data on the effect of LAB on the β-glucan content of cereal-based substrates.

Thus, the present study was aimed at exploring a large pool of cereal-sourced LAB for their potential use as sourdough starter cultures for the baking industry, with a specific focus on the following–separate or combined—target applications, namely (i) improvement of digestibility and mineral availability of wholegrain high-phytate bread; (ii) improvement of bread softness and sensory perception; (iii) enhancement of bread health-promoting properties.

To this end, 152 LAB were preliminarily screened for the production of phytases and EPS on the appropriate solid media. The EPS-producing isolates were molecularly screened for the occurrence of key genes involved in the synthesis of EPS. In parallel, the molecular weights and monomer compositions of the EPS purified from the selected pool of isolates were determined. Finally, the effect of fermentation by the selected LAB on the β-glucan content of an *ad hoc* formulated barley flour medium was evaluated.

## Materials and methods

### Lactic acid bacteria isolation

One hundred and fifty-two LAB isolates were subjected to an *in vitro* pro-technological and functional characterization. Among these, 121 LAB had previously been isolated from: i) soft wheat sourdoughs used for bread-making at 8 local artisan bakeries (93 isolates, labeled as PB) [24]; ii) a soft wheat sourdough produced with stone grinded wholemeal wheat flour (10 isolates, labeled as LM) [25]; iii) a soft wheat sourdough used for production of recurrence sweet leavened cakes at a local industrial bakery (3 isolates, labeled as LD) [26]; and iv) commercial Boza, an unpasteurized fermented cereal-based beverage (15 isolates, labeled as Bz) [27]. The remaining 31 LAB were isolated in the present study from wholemeal wheat (1 isolate, labeled as FG, emmer (23 isolates, labeled as FF), and barley (7 isolates, labeled as FO) flours purchased from a local mill (Luzi S.r.l., Sassoferrato, Italy). All the 152 isolates were maintained at the Culture Collection of the Department of Agriculture, Food and Environmental Science (Università Politecnica delle Marche, Ancona, Italy) at -80°C in a mixture of glycerol and MRS broth (VWR International, Milan, Italy), at a 1:1 ratio. They were subcultured on MRS agar incubated at 30°C for 48 h prior to further analysis.

The DNA was extracted, as previously described by Osimani et al. [27], from the 31 newly isolated flour sourced LAB and the 13 still unidentified isolates (labeled as LM and LD, respectively). The extracted DNA was quantified, checked for the purity using a NanoDrop ND 1000 (Thermo Fisher Scientific, Wilmington, DE, USA) and normalized to a concentration of 25 ng μL^-1^. Two μL aliquots of the extracts (containing approximately 50 ng of DNA) were amplified by PCR in a My Cycler Thermal Cycler (BioRad Laboratories, Hercules, CA, USA) using the universal eubacterial primer pair 27F and 1495R [28]. The amplification reactions were carried out according to the conditions previously described by Osimani et al. [27]. The PCR products were checked on 1.5% (w v^-1^) agarose gels and sent to Genewiz (Takeley, UK) for purification and sequencing. The obtained raw sequences in FASTA format were checked for chimeras using Uchime software package [29], whereas terminal NNNs and misleading data from the ends of the sequences were trimmed before further analysis. Hence, the processed sequences were compared with 16S rRNA sequences of type strains deposited in the GenBank DNA database (http://www.ncbi.nlm.nih.gov/) using the Basic Local Alignment Search Tool (BLAST) [30]. The sequences of the newly identified isolates were deposited in the GenBank DNA database under accession numbers MT501108- MT501151. A detailed list of the 152 molecularly identified isolates is reported in S1 Table.

### Phytase activity

All the 152 LAB isolates were screened for their ability to degrade phytic acid on slightly modified Sourdough Simulation Medium (SSM) [31] containing: fructose (10 g L^-1^), maltose (10 g L^-1^), glucose (2 g L^-1^), tryptone (10 g L^-1^), yeast extract (12 g L^-1^), L-cysteine HCl (0.5 g L^-1^), MgSO_4_ X 7 H_2_O (0.2 g L^-1^), MnSO_4_ X H_2_O (0.05 g L^-1^), and Tween 80 (1 mL L^-1^).

Phytase activity was evaluated by the phytase plate assay using a combination of the methods previously described by Anastasio et al. [32], Fossi et al. [33], Manini et al. [34] and Raghavendra and Halami [35], respectively. The isolates were first grown at 37°C for 48 h in SSM broth supplemented with sodium phytate (1%, w v^-1^) (Merck Life Science S.R.L., Milan, Italy); 3 μL of each broth culture was spotted (in duplicate) with a manual pipette on 90 mm plates containing SSM supplemented with sodium phytate (1%, w v^-1^), CaCl_2_ (0.2%, w v^-1^) and agar (18 g L^-1^). For each isolate, three spots were produced on the same plate. The plates, each including six spots (from two isolates), were incubated at 37°C for 48 h and further examined for the appearance of clearance zones around the spots. To eliminate false positives, after incubation, the colonies were washed off from the agar surface using distilled water. The plates were then flooded with a 2% (w v^-1^) aqueous cobalt chloride solution for 5 min at room temperature and subsequently counterstained with a 6.25% (w v^-1^) ammonium molybdate solution for 5 min at room temperature. The phytase activity was evaluated by measuring the diameter (in mm) of the clear halos surrounding the spots. The isolates were grouped in three arbitrary classes, based on the mean diameter of their clear halos: class 1 (labeled as +++), with a halo diameter ranging from 21 to 27 mm; (ii) class 2 (labeled as ++), with a halo diameter ranging from 11 and 20 mm; (iii) and class 3 (labeled as +), with a halo diameter ranging from 4 to 10 mm.

### EPS production

The pool of 152 LAB were further screened for the ability to produce EPS. The LAB were first grown in SSM broth incubated at 30°C for 48 h. An aliquot of 5 μL of each broth culture was spotted (in duplicate) on 90 mm plates containing SSM agar supplemented with only sucrose (5%, w v^-1^) as a replacement for fructose, maltose, and glucose. For each isolate, three spots were produced on the same plate. The plates, each including six spots (from two isolates), were incubated at 30°C for 72 h. After the incubation period, the colonies were classified as “mucoid” or “ropy”, depending on their appearance and slime production. Mucoid colonies had a slimy and glistening appearance but did not produce long visible filaments when picked with a sterile toothpick. Ropy colonies were grouped into the following three arbitrary classes, based on the mean length of the filaments picked from the colony: class 1 (labeled as +++), with a filament mean length ≥ 11 mm; class 2 (labeled as ++), with a mean length ranging from 6 to 10 mm; and class 3 (labeled as +) with a mean length ranging from 3 to 5 mm.

### EPS extraction

The best EPS-producing LAB (13 isolates, including all the ropy colonies grouped in class 1 and 2 and some randomly selected mucoid colonies) were selected for determination of the monosaccharide composition and molecular weight of the produced EPS. For preliminary EPS extraction, each isolate was grown in SSM broth incubated at 30°C for 24 h, and the bacterial biomass was harvested by centrifugation at 4000 rpm for 5 min. Once the supernatant was discarded, each cell pellet was resuspended in a sterile physiological solution (0.85% NaCl, w v^-1^), and the concentration of LAB cells was determined spectrophotometrically at 600 nm using a UV-Vis Shimadzu UV-1800 spectrophotometer (Shimadzu Corporation, Kyoto, Japan). Cell suspensions were standardized to a final concentration of approximately 5 x 10^7^ cells mL^-1^ using a sterile physiological solution; an aliquot (500 μL) of each suspension was streaked (in duplicate) on 90 mm plates containing SSM agar supplemented with sucrose (5%, w v^-1^). After incubation at 30°C for 48 h, the EPS produced by each LAB isolate were washed off from the two plates by sterile deionized water, pooled together and further processed as described by Palomba et al. [36].

### EPS monosaccharide composition

Purified EPSs were hydrolysed according to Zhou et al. [37]. Monosaccharides were than derivatized (silylated) according to Lucci et al. [38] and analyzed on a CP-9001 gas chromatograph (Chrompack, Middelburg, The Netherlands) equipped with a column TG-17Sil MS, 30 m length × 0.25 mm i.d., 0.25 μm film thickness (Thermo Fisher Scientific, MA, USA). Helium was used as carrier gas at a linear rate of 35 cm/s. The oven temperature was set at 150°C for 2 min, then increased at a rate of 5°C min^-1^ to the final temperature of 270°C, which was kept for 10 min. Injector and flame ionization detector (FID) temperatures were set at 270°C.

### EPS molecular weight

The average molecular weight of the purified EPSs was estimated by gel-permeation chromatography. An Agilent 1100 Series quaternary pump (Agilent Technologies, CA, USA) coupled with a Jasco 880 RI detector (Jasco Europe S.r.l., Italy) was used. Separation was carried out using the column PROGEL-TSK GMPWXL, 7.8 x 300 mm (Tosoh Bioscience, Japan), according to the conditions described by Zhou et al. [37]. Commercial dextrans (5, 12, 50, 80, and 410 KDa) from *Leuconostoc*. *mesenteroides* (Merck KGaA, Darmstadt, Germany) were used for the calibration of the chromatographic system. A linear regression Log molecular weight vs retention time was used to estimate the dextran-equivalent molecular weights of the purified EPS.

### Molecular detection of EPS-related genes

The LAB isolates producing EPS on SSM agar supplemented with sucrose (5%, w v^-1^) were further screened for the presence of the genes *gtf* (coding for glucansucrase) and *lev* (coding for levansucrase) and for the genes *eps*A, *eps*B, *eps*D/E plus *eps*EFG, which are involved in HoPS and HePS production, respectively. The DNA was extracted from these isolates, performed as previously described by Osimani et al. [27], and amplified by PCR using the primer pairs and thermal cycling conditions previously reported by Palomba et al. [36]. Briefly, 3 μL of each DNA extract (containing about 75 ng of bacterial DNA) was amplified in a 50 μL-reaction mixture containing 1 μM of each primer and 25 μL of Green PCR Master Mix Direct-Load 2X (Biotechrabbit, Hennigsdorf, Germany). For each gene under study, amplicons from a few arbitrarily selected PCR-positive isolates were sent to Genewiz for purification and sequencing to confirm the reaction specificity. The obtained sequences in FASTA format were aligned with those deposited in the GenBank database (http://www.ncbi.nlm.nih.gov/) using the Basic Local Alignment Search Tool (BLAST) [30].

### Effect of LAB on β-glucan content

The LAB carrying the *gtf* gene were further assayed in duplicate for their interaction with the β-glucans contained in a sterilized barley flour (BF) medium, prepared by adding 5% barley flour (w v^-1^) to deionized water. Each isolate was inoculated in tubes containing 10 mL of BF medium to reach a final cell concentration of approximately 2 x 10^8^ cells mL^-1^. The β-glucan content of the liquid medium was determined immediately before inoculation and after 24 h of fermentation at 30°C using a commercial mixed-linkage beta-glucan kit (Megazyme, Bray, Ireland) according to the assay procedure suggested by the kit manufacturer for liquid samples (including alcohol precipitation). The results were expressed as the mean variance (%) (positive or negative) of the β-glucan content before and after fermentation ± standard deviation.

### Statistical analysis

The values of β-glucan content before and after fermentation were subjected to one-way analysis of variance (ANOVA), whereas the significant differences in the EPS monosaccharide composition between different isolates were analyzed using the Tukey-Kramer’s Honest Significant Difference test. All statistical analyses were performed using the JMP statistical software, version 11.0.0 (SAS Institute Inc., Cary, NC, USA) with criterion of significance set at P < 0.05.

## Results and discussion

### Molecular identification of LAB

The results of the 16S rRNA sequencing of the 31 LAB isolated in this study from wheat, emmer, and wholegrain barley flours (labeled as FG, FF and FO, respectively) and of those previously isolated from sourdoughs (labeled as LD and LM) are reported in Table 1. The 16S rRNA gene sequencing is widely used for the identification and classification of bacteria. This method relies on the comparison of the 16S rRNA gene sequence of an unknown isolate against the deposited sequences of bacterial type or reference strains. Recently, a 98.65% cut-off has been set for the identification of species [39]. Though 16S rRNA gene sequencing is recognized as one of the most efficient molecular techniques for the establishment of taxonomic relationships between prokaryota, it is not suitable for the discrimination of some closely related species, as those belonging to the *Lactobacillus casei*, *Lactobacillus plantarum*, *Lactobacillus buchneri*, and *Lactobacillus sakei* groups, which are characterized by a high-level similarity (≥ 99%) in the 16S rRNA gene sequences. This implies that advanced methods, including whole genome sequencing or multilocus sequence typing (MLST), can be applied to differentiate these microorganisms [40].

**Table 1:** 16S rRNA identification of lactic acid bacteria (LAB) isolated in the present study from different flours and sourdoughs.

Isolate code	Species	Isolation source	% Identity^a^	Accession number^b^	Accession number^c^
FF2	*Lactobacillus curvatus Lactobacillus graminis*	emmer flour	99.42 99.42	MT501108	NR_113334^T^ NR_042438^T^
FF3	*Lactobacillus curvatus*	emmer flour	99.06	MT501109	NR_113334^T^
FF5	*Lactobacillus curvatus*	emmer flour	99.32	MT501110	NR_113334^T^
FF7	*Lactobacillus curvatus Lactobacillus graminis*	emmer flour	99.18 99.18	MT501111	NR_113334^T^ NR_042438^T^
FF15	*Lactobacillus coryniformis*	emmer flour	98.82	MT501112	NR_029018^T^
FF33	*Lactobacillus curvatus*	emmer flour	99.71	MT501113	NR_113334^T^
FF41	*Enterococcus durans*	emmer flour	99.88	MT501114	NR_113900^T^
FF42	*Enterococcus durans*	emmer flour	99.88	MT501115	NR_113900^T^
FF43	*Lactobacillus curvatus*	emmer flour	99.63	MT501116	NR_113334^T^
FF44	*Lactobacillus curvatus*	emmer flour	99.63	MT501117	NR_113334^T^
FF45	*Enterococcus durans*	emmer flour	99.46	MT501118	NR_113900^T^
FF46	*Enterococcus durans*	emmer flour	99.37	MT501119	NR_113900^T^
FF48	*Lactobacillus curvatus*	emmer flour	99.57	MT501120	NR_113334^T^
FF49	*Enterococcus durans*	emmer flour	99.56	MT501121	NR_113900^T^
FF50	*Enterococcus durans*	emmer flour	99.13	MT501122	NR_113900^T^
FF51	*Lactobacillus curvatus Lactobacillus graminis*	emmer flour	99.57 99.57	MT501123	NR_042437^T^ NR_042438^T^
FF52	*Lactobacillus curvatus Lactobacillus graminis*	emmer flour	99.57 99.57	MT501124	NR_042437^T^ NR_042438^T^
FF53	*Lactobacillus curvatus Lactobacillus graminis*	emmer flour	99.64 99.64	MT501125	NR_042437^T^ NR_042438^T^
FF54	*Enterococcus durans*	emmer flour	99.54	MT501126	NR_113257^T^
FF71	*Pediococcus pentosaceus*	emmer flour	99.53	MT501127	NR_042058^T^
FF78	*Pediococcus pentosaceus*	emmer flour	99.28	MT501128	NR_042058^T^
FF86	*Pediococcus pentosaceus*	emmer flour	99.35	MT501129	NR_042058^T^
FF95	*Pediococcus pentosaceus*	emmer flour	99.36	MT501130	NR_042058^T^
FO2	*Lactobacillus plantarum*	barley flour	99.15	MT501132	NR_115605^T^
FO8	*Pediococcus pentosaceus*	barley flour	99.23	MT501133	NR_042058^T^
FO13	*Lactobacillus plantarum*	barley flour	99.06	MT501134	NR_115605^T^
FO27	*Pediococcus pentosaceus*	barley flour	99.40	MT501135	NR_042058^T^
FO30	*Pediococcus pentosaceus*	barley flour	99.60	MT501136	NR_042058^T^
FO40	*Pediococcus pentosaceus*	barley flour	99.51	MT501137	NR_042058^T^
FO41	*Pediococcus pentosaceus*	barley flour	99.79	MT501138	NR_042058^T^
FG2	*Enterococcus casseliflavus*	wheat flour	98.92	MT501131	NR_119280^T^
LM1	*Lactobacillus paralimentarius*	sourdough	99.31	MT501139	NR_114844^T^
LM2	*Lactobacillus paralimentarius*	sourdough	99.32	MT501140	NR_114844^T^
LM3	*Lactobacillus paralimentarius*	sourdough	99.51	MT501141	NR_114844^T^
LM4	*Lactobacillus paralimentarius*	sourdough	99.51	MT501142	NR_114844^T^
LM5	*Lactobacillus paralimentarius*	sourdough	99.57	MT501143	NR_114844^T^
LM6	*Lactobacillus paralimentarius*	sourdough	99.32	MT501144	NR_114844^T^
LM7	*Lactobacillus paralimentarius*	sourdough	99.14	MT501145	NR_114844^T^
LM8	*Lactobacillus paralimentarius*	sourdough	99.30	MT501146	NR_114844^T^
LM9	*Lactobacillus brevis*	sourdough	99.88	MT501147	NR_116238^T^
LM10	*Lactobacillus paralimentarius*	sourdough	99.68	MT501148	NR_114844^T^
LD58	*Lactobacillus brevis*	sourdough	99.90	MT501149	NR_116238^T^
LD65	*Lactobacillus brevis*	sourdough	100.00	MT501150	NR_116238^T^
LD66	*Lactobacillus brevis*	sourdough	100.00	MT501151	NR_116238^T^

^a^ Percentage of identical nucleotides in the 16S rRNA sequence obtained from the LAB isolates and the sequence of the closest relative (type strain) from the GenBank database.

^b^ GenBank accession number of the deposited sequences

^c^ GenBank accession number of the sequence of the closest relative (type strain) found by BLAST search

^T^Type strain

Flour-sourced LAB were identified as *Lactobacillus curvatus/Lactobacillus graminis* (11 isolates), *Enterococcus durans* (7 isolates), *Pediococcus pentosaceus* (4 isolates) and *Lactobacillus coryniformis* (1 isolate) based on BLAST (megablast algorithm) analysis of their 16S rRNA sequences. For all the isolates analyzed, except for FF2, FF7, FF51, FF52 and FF53 (accession numbers MT501108, MT501111, MT501123, MT501124 and MT501125, respectively), showing the same percent identity with *Lb*. *curvatus* and *Lb*. *graminis*, an unambiguous identification was achieved.

**Fig 1:**
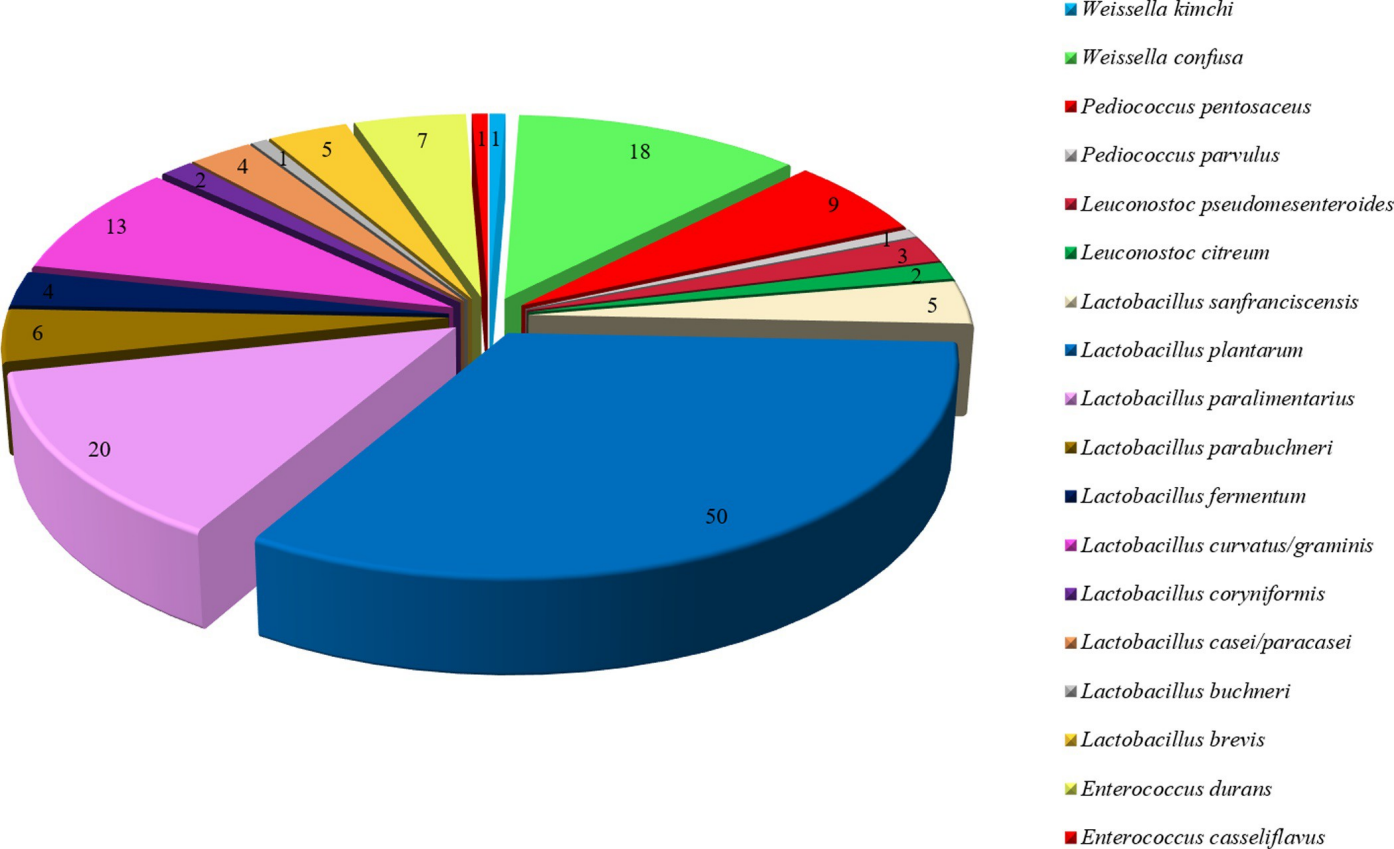
Species distribution of the 152 lactic acid bacteria (LAB) analyzed in this study (for each sector, the number of isolates is reported).

The species *Lb*. *curvatus* and *P*. *pentosaceus* have previously been detected in emmer flour [41, 42]. The seven LAB isolated from wholegrain barley flour were identified as *P*. *pentosaceus* (5 isolates) and *Lb*. *plantarum* (2 isolates). Both these species have previously been isolated from barley sourdoughs [43], where the latter was found to dominate after two-month refreshments [44, 45]. The sole LAB isolate obtained from wheat flour was ascribed to *Enterococcus casseliflavus*. As reported by Corsetti et al. [46], this species is frequently isolated from wheat grains, bran, and non-conventional flours. Finally, the 13 sourdough-sourced isolates were ascribed to *Lactobacillus brevis* (4 isolates) and *Lactobacillus paralimentarius* (9 isolates), with both species being recognized as typical sourdough LAB [47]. The remaining 108 isolates screened in this study had previously been identified by Osimani et al. [24, 27]. In Fig 1, a complete picture of the 152 members of the LAB pool is depicted; as a whole, 18 species ascribed to four genera were included, namely: (i) *Lactobacillus* (*Lb*. *brevis*, *Lb*. *buchneri*, *Lactobacillus casei/paracasei*, *Lb*. *coryniformis*, *Lb*. *curvatus/graminis*, *Lactobacillus fermentum*, *Lactobacillus parabuchneri*, *Lb*. *paralimentarius*, *Lb*. *plantarum*, *Lactobacillus sanfranciscensis*); (ii) *Leuconostoc* (*Leuconostoc citreum*, *Leuconostoc pseudomesenteroides*); (iii) *Pediococcus* (*Pediococcus parvulus*, *P*. *pentosaceus*); *Weissella (Weissella confusa*, *Weissella kimchi*); and (iv) *Enterococcus* (*E*. *casseliflavus*, *E*. *durans*). A detailed list of the LAB with their source of isolation is reported in the S1 Table.

### Phytase activity

The results of the phytase activity screening carried out on SSM agar (supplemented with sodium phytate and CaCl_2_) used to simulate the typical sourdough ecological environment are shown in Table 2. More details are reported in the S1 Table. The majority (143 out of 152) of the screened LAB were able to degrade sodium phytate on the solid medium used. This finding agrees well with those reported in the study performed by Manini et al. [34], where 12 out of 13 LAB isolated from a spontaneously fermented wheat bran sourdough were able to degrade sodium phytate. In another study, the majority (71.6%) of 88 LAB isolated from *atole agrio*, a fermented Mexican maize-based beverage, degraded phytate [48]. Similarly, De Angelis et al. [49] reported that 8 out of 13 sourdough-sourced LAB could degrade both sodium and hexacalcium phytate. A different picture emerged from the study of Raghavendra and Halami [35], where the 40 LAB assayed were able to hydrolyze hexacalcium phytate, but only two of these isolates (*P*. *pentosaceus* CFR R38 and CFR R35) were able to degrade sodium phytate. However, when CaCl_2_ was added to modified MRS agar supplemented with phytate salt (calcium or sodium), all the assayed LAB hydrolyzed sodium phytate, thus indicating a possible involvement of calcium ions in this enzymatic activity.

**Table 2:** Phytase activity of lactic acid bacteria (LAB) isolates determined using phytase plate assay.

Species	Isolate codes			
	Phytase activity (arbitrary classes*)			
	+++	++	+	-
*Enterococcus casseliflavus*		FG2		
*Enterococcus durans*		FF46, FF45	FF41, FF42, FF49, FF50, FF54	
*Lactobacillus brevis*	LD65, LD66	PB57, LD58, LM9		
*Lactobacillus buchneri*	Bz32			
*Lactobacillus casei/paracasei*		PB294, Bz33, Bz34	Bz10, Bz35	
*Lactobacillus coryniformis*		FF15	Bz44	
*Lactobacillus curvatus/graminis*	FF33, FF43	FF48, FF5, FF7, FF52, FF44, FF53	PB1, PB115, FF2, FF3, FF51	
*Lactobacillus fermentum*	Bz5	PB160, Bz2, PB162		
*Lactobacillus parabuchneri*		Bz38, Bz26, Bz36, Bz31, Bz37, Bz28		
*Lactobacillus paralimentarius*		PB232, PB125, PB233, PB126, PB231, PB94, LM5, PB230, PB127	PB229, LM3, LM10, LM2, LM4, LM6, LM7, LM8, PB265, LM1	
*Lactobacillus plantarum*	PB241, PB97, PB173, PB268, PB305, FO2, PB15, PB11, PB278, PB308	PB98, FO13, PB85, PB306, PB275, PB24, PB257, PB277, PB307, PB191, PB151, PB84, PB190, PB199, PB202, PB242, PB296, PB297, PB298, PB304, PB46, PB240, PB256, PB181, PB193, PB22, PB86, PB124, PB210, PB14, PB134, PB200, PB239, PB105, PB209, PB255, PB104, PB287	PB128, PB161	
*Lactobacillus sanfranciscensis*	PB219	PB221, PB223, PB276		PB213
*Leuconostoc citreum*		PB220	PB3	
*Leuconostoc pseudomesenteroides*		PB295	PB170	PB288
*Pediococcus parvulus*			Bz39	
*Pediococcus pentosaceus*	FF86, FO40	FO27, FO30, FF95, FO41, FO8, FF71	FF78	
*Weissella confusa*		PB309, PB311, PB152	PB321, PB327, PB323, PB324, PB330, PB331, PB334, PB313	PB314, PB315, PB319, PB325, PB332, PB333, PB338
*Weissella kimchi*		PB337		

*Arbitrary classes were defined on the basis of the mean diameter of the clear halo surrounding the colonies: +++ (class 1), 21–27 mm; ++ (class 2), 11–20 mm, + (class 3), 4–10 mm; -, no clear halo

In regard to the diameter of the clear haloes, mean values between 4 and 27 mm were recorded, thus revealing the high heterogeneity in the phytase activity of the 152 LAB, even within the same species. The isolates classified as class 1 (+++) and hence producing the largest clear halos (with a mean diameter of 21–27 mm) belonged to the following species: *Lb*. *brevis*, *Lb*. *buchneri*, *Lb*. *curvatus*, *Lb*. *fermentum*, *Lb*. *plantarum*, *Lb*. *sanfranciscensis*, and *P*. *pentosaceus*. Among these isolates, *Lb*. *plantarum* PB241 and *Lb*. *brevis* LD65 showed the highest phytase activity, with a mean halo diameter of 27 mm. Of note, most of the isolates belonging to the latter two species showed a high or intermediate phytase activity, with only 2 out of 50 *Lb*. *plantarum* isolates being classified as weak phytase producers (class 3, +). Similarly, in a recent study by Sümengen et al. [50], *Lb*. *brevis* isolated from Hatay boiled cheese produced high levels of both extracellular and intracellular phytases. To the authors’ knowledge, to date, most of the phytase activity assays carried out on LAB have used solid media other than SSM agar, thus making the comparison of our data with those available in the literature cumbersome. In this regard, Anastasio et al. [32] reported clear halos ranging from 3 to 13 mm on modified Chalmers agar supplemented with 1% hexacalcium phytate, with a *L*. *plantarum* strain isolated from pizza dough producing a 13 mm-diameter clear halo. The study performed by Manini et al. [34] reported even smaller halo diameters (1–3 mm) on the same medium, except for the *Lb*. *brevis*, *Lb*. *plantarum* and *L*. *citreum* strains producing clear halos with diameters larger than 3 mm. In the present study, the majority of *P*. *pentosaceus* isolates showed high (+++) or intermediate (++) phytase activity, with the two isolates FF86 and FO40 collected from emmer and barley flour, respectively, both producing 21 mm clear halos. The latter finding agrees well with what was reported by Cizeikiene et al. [51], where the two strains *P*. *pentosaceus* KTU05-8 and KTU05-9 were recommended as suitable starters for the preparation of wholemeal wheat sourdough, due to their high phytase activity. More recently, Rizzello et al. [52] reported that in fava beans fermented by the strain *P*. *pentosaceus* VTTT E-153483, the phytic acid content was reduced to more than half the initial concentration. Among the five *Lb*. *sanfranciscensis* isolates assayed in the present study (PB213, PB219, PB221, PB223 and PB276), only the first showed no phytase activity on SSM agar, whereas the remaining 4 produced clear halos with diameters between 14 mm and 21 mm. Again, this finding agrees well with those reported in a study performed by De Angelis et al. [49], where *Lb*. *sanfranciscensis* strains showed a high phytase activity on sodium phytate. As far as the isolates ascribed to *W*. *confusa* are concerned, most of them had low (+) or no phytase activity (-). For this species, a very different picture emerged from the study of Mandhania et al. [53], where all the strains assayed produced phytases.

### EPS production

Table 3 reports the results of the LAB screening for EPS production on SSM agar supplemented with 5% sucrose. The visual inspection of colonies grown on a solid medium is one of the most frequently used methods for the screening of EPS-producing LAB, being particularly suitable for the detection of high EPS producers [15]. EPS production mainly depends on the composition of the growth medium in terms of carbon and nitrogen sources, as well as on other parameters, including the pH of the growth medium, incubation temperature and time. Palomba et al. [36] clearly demonstrated that a growth medium supplemented with sucrose is more suitable for screening EPS-producing LAB compared to the same growth medium supplemented with other carbohydrate sources, such as maltose, glucose, galactose, lactose, and fructose. As shown in Table 3, only 28 out of the assayed 152 LAB (18%) were able to produce EPS. This finding is consistent with the results reported in the study carried out by Palomba et al. [36], where 34 out of 177 LAB (19%) isolated from doughs and sourdoughs were able to produce EPS on modified Chalmers agar supplemented with sucrose.

**Table 3:** Screening of lactic acid bacteria (LAB) for the exopolysaccharides (EPS) production on Sourdough Simulation Medium (SSM) added with sucrose (5%, w v^-1^), the genes involved in their production, and the variance of β-glucan content in barley flour medium before and after the fermentation.

Isolate code	Species	Source	EPS production	EPS genes						β-glucans (%)^a^
				*eps*A	*eps*B	*eps*D/E	*eps*EFG	*gtf*	*lev*	
**LD58**	*Lactobacillus brevis*	sourdough	R (++)	+	-	+	-	-	-	n.d.
**LD65**	*Lactobacillus brevis*	sourdough	R (+)	+	-	+	-	-	-	n.d.
**LD66**	*Lactobacillus brevis*	sourdough	R (+++)	+	-	+	-	-	-	n.d.
**FF7**	*Lactobacillus curvatus/graminis*	emmer flour	R (+)	+	-	-	-	-	-	n.d.
**Bz2**	*Lactobacillus fermentum*	boza	R (++)	-	-	+	-	-	-	n.d.
**Bz5**	*Lactobacillus fermentum*	boza	R (+)	-	-	-	-	-	-	n.d.
**PB127**	*Lactobacillus paralimentarius*	sourdough	M	-	-	+	-	-	-	n.d.
**PB181**	*Lactobacillus plantarum*	sourdough	M	-	-	+	-	-	-	n.d.
**PB3**	*Leuconostoc citreum*	sourdough	M	-	-	+	-	+	-	3.43 ± 0.74*
**PB220**	*Leuconostoc citreum*	sourdough	M	-	-	+	-	+	-	6.86 ± 0.37*
**PB170**	*Leuconostoc pseudomesenteroides*	sourdough	M	-	-	+	-	-	-	n.d.
**PB288**	*Leuconostoc pseudomesenteroides*	sourdough	R (+)	-	-	-	-	-	-	n.d.
**FF71**	*Pediococcus pentosaceus*	emmer flour	R (++)	-	-	+	-	-	-	n.d.
**FF78**	*Pediococcus pentosaceus*	emmer flour	R (++)	-	-	+	-	-	-	n.d.
**FF95**	*Pediococcus pentosaceus*	emmer flour	R (+)	-	-	-	-	-	-	n.d.
**FO8**	*Pediococcus pentosaceus*	barley flour	R (+)	-	-	+	-	-	-	n.d.
**FO27**	*Pediococcus pentosaceus*	barley flour	R (+++)	-	-	-	-	-	-	n.d.
**FO30**	*Pediococcus pentosaceus*	barley flour	R (++)	-	-	-	-	-	-	n.d.
**PB315**	*Weissella confusa*	sourdough	M	-	-	+	-	+	-	-1.37 ± 0.49
**PB323**	*Weissella confusa*	sourdough	M	+	-	+	-	+	-	4.93 ± 2.28
**PB324**	*Weissella confusa*	sourdough	M	-	-	+	-	+	-	-1.15 ± 1.13
**PB325**	*Weissella confusa*	sourdough	M	-	-	-	-	-	-	n.d.
**PB327**	*Weissella confusa*	sourdough	M	-	-	+	-	+	-	1.08 ± 1.08
**PB330**	*Weissella confusa*	sourdough	M	-	-	-	-	+	-	3.94 ± 0.87*
**PB331**	*Weissella confusa*	sourdough	M	-	-	-	-	+	-	4.66 ± 1.00*
**PB332**	*Weissella confusa*	sourdough	M	+	-	-	-	+	-	7.32 ± 1.08*
**PB333**	*Weissella confusa*	sourdough	M	+	-	+	-	+	-	6.45 ± 0.69*
**PB337**	*Weissella confusa*	sourdough	M	+	-	+	-	+	-	3.29 ± 2.65

R: Ropy colonies, in brackets the arbitrary class defined on the basis of the length of the EPS filaments originated from the colonies [+++ (class 1), ≥ 11 mm; ++ (class 2), 6–10 mm; + (class 3), 3–5 mm]; M: mucoid colonies; EPS genes: +, presence of the corresponding gene; -, no detection of the corresponding gene;

^a^the mean (%) variance (positive or negative) of β-glucan content in barley flour medium before and after the fermentation ± standard deviation;

*, significantly different (P < 0.05); n.d., not determined

In the present study, 13 and 15 isolates out of the 28 EPS-producing LAB produced ropy and mucoid colonies, respectively, with the first group including isolates ascribed to *Lb*. *brevis*, *Lb*. *curvatus/graminis*, *Lb*. *fermentum*, and *P*. *pentosaceus*. Among these ropy isolates, the colonies formed by *Lb*. *brevis* LD66 (sourced from sourdough) and by *P*. *pentosaceus* FO27 (sourced from wholegrain barley flour) produced the longest filaments (mean length ≥ 11 mm) when picked with a sterile toothpick. Most of the isolates ascribed to the above listed species were good EPS producers. In more detail, six out of the nine *P*. *pentosaceus* isolates assayed were able to produce EPS on SSM agar. This finding is consistent with the study performed by Smitinont et al. [54], where LAB isolated from various fermented foods were screened for their EPS production, with *P*. *pentosaceus* strains showing particularly high EPS yields. The role of *P*. *pentosaceus* in EPS synthesis was further investigated by Abedfar et al. [55], who found that most of the LAB isolates from wheat bran sourdough ascribed to *P*. *pentosaceus* were EPS producers.

As expected, three out of the five *Lb*. *brevis* isolates assayed in the present study produced EPS on SSM agar. In fact, among LAB, *Lb*. *brevis* is widely recognized as one of the major EPS producers [9]. As early as 1995, Figueroa et al. [56] reported that this species produces ropy colonies on glucose- or sucrose-containing media.

The remaining two species producing exclusively ropy colonies were *Lb*. *curvatus/graminis* and *Lb*. *fermentum*. Regarding the latter species, two (Bz2 and Bz5) out of the four isolates sourced from Boza were able to form colonies producing 5- to 8-mm filaments when picked. To date, EPS production by *Lb*. *fermentum* has been widely documented [9, 57]. Concerning *Lb*. *curvatus/graminis*, only one (FF7) out of the 13 isolates assayed (sourced from wholegrain emmer flour) produced EPS. Minervini et al. [58] have previously reported a highly efficient synthesis of EPS by the strain *Lb*. *curvatus* DPPMA10 during its growth on hydrolyzed wheat flour agar.

Regarding *L*. *pseudomesenteroides*, two out of three isolates tested in this study synthesized EPS, with the isolates PB288 and PB170 being characterized by a ropy and mucoid EPS phenotype, respectively. To date, numerous studies have established the EPS-producing behavior of *L*. *pseudomesenteroides*, as shown for some strains isolated from Turkish sourdoughs [59] or soybean paste [60]. Moreover, Du et al. [61] purified and characterized dextran, an exopolysaccharide synthesized by the strain *L*. *pseudomesenteroides* DRP-5, with a moderate antioxidant activity in *in vitro* experiments. Among the other properties of dextrans produced by *L*. *pseudomesenteroides*, the polymer produced by the strain YB-2 showed a high water solubility index, a water retention capacity and inhibitory effects against pathogenic microorganisms such as *Escherichia coli* and *Staphylococcus aureus* [62].

In the present study, all the isolates belonging to *Lb*. *paralimentarius*, *Lb*. *plantarum*, *L*. *citreum*, and *W*. *confusa* produced mucoid colonies. According to De Vuyst and De Vin [63], this EPS phenotype is associated with the production of extracellular HoPS. In more detail, only one (PB127) out of the twenty *Lb*. *paralimentarius* sourdough isolates analyzed were able to produce EPS. This finding agrees well with those reported by Dertli et al. [59], who found a very limited capacity of producing EPS by some sourdough *Lb*. *paralimentarius* isolates.

For *L*. *citreum*, the two assayed sourdough isolates showed a mucoid EPS phenotype, analogous to the strain *L*. *citreum* L3C1E7 isolated from Pico cheese by Domingos-Lopes et al. [64].

Regarding *W*. *confusa*, 10 out of the 18 assayed isolates were EPS producers. In 2015, EPS produced by *W*. *confusa* were analyzed by Tinzl-Malang et al. [65], who explored the use of members of this species to improve the quality of bakery products, specifically bread, both in terms of shelf life and texture. In a more recent study [66], the strain *W*. *confusa* QS813, isolated from a type I sourdough, synthesized high molecular weight dextrans with a positive effect on hydration, water distribution and microstructure of gluten during the freeze-thaw process.

Surprisingly, only one (PB181) out of the fifty *Lb*. *plantarum* isolates screened in this study produced EPS on SSM agar. This finding was quite unexpected, given the numerous available reports on the EPS produced by *Lb*. *plantarum*, which are known to have antitumor, antioxidant and antibiofilm activities. The EPS synthesized by *Lb*. *plantarum* have also been exploited by the food industry as emulsifiers, gelling agents and stabilizers, as well as texture improvers in fermented foods [67].

### Molecular detection of EPS-related genes

Twenty-eight LAB showing a ropy or mucoid phenotype on SSM agar supplemented with sucrose (5%, w v^-1^) were further assayed for the occurrence of key genes involved in the synthesis of both HoPS (e.g., *gtf* and *lev*) and HePS (e.g., *eps*A, *eps*B, *eps*D/E and *eps*EFG). The results of the molecular screening are reported in Table 3. The sequencing of the amplicons from randomly selected PCR-positive isolates confirmed the specificity of all the amplification protocols used.

Twenty-two out of the 28 tested isolates harbored at least one of the EPS-related genes of interest. Some LAB belonging to *L*. *citreum* and *W*. *confusa* harbored both HoPS- and HePS-related genes, as previously reported by Van der Meulen et al. [68] and Palomba et al. [36]. In the present study, the glycosyltransferase genes e*ps*D/E were found with the highest frequency (18 out of 22 screened isolates). Among the two EPS-producing isolates ascribed to *Lb*. *fermentum*, only one (Bz2) carried *eps*D/E. The occurrence of the genes involved in EPS synthesis in *Lb*. *fermentum* has recently been documented by Harris et al. [69], who sequenced the whole genome of the strain *Lb*. *fermentum* Lf2, highlighting 3 gene clusters potentially involved in the EPS production process. In regards to *P*. *pentosaceus*, three (FF71, FF78 and FO8) out of the six EPS-producing isolates assayed harbored *eps*D/E, while the remaining three isolates, including FO27, one of the best EPS producers, were negative for all the tested genes. This could be explained by a possible variation in one or both the primer binding sites of the EPS genes, which in turn might have hindered the successful amplification of these target genes. An alternative explanation relies on the involvement of other genes in EPS synthesis, as suggested by a recent investigation [70] on the EPS-biosynthetic gene cluster of a *P*. *pentosaceus* strain (LP28) isolated from *Euphoria longana*, where 12 open reading frames (ORFs) containing a priming enzyme, five glycosyltransferases, and a putative polysaccharide pyruvyl transferase were identified.

All the *Lb*. *brevis* EPS-producing isolates on SSM agar harbored *eps*D/E and *eps*A, thus confirming the results previously reported by Dertli et al. [59], whereas the strain *Lb*. *brevis* E-25 harbored *eps*D/E, *eps*A, *eps*EFG and *gtf*. The *eps*D/E gene was also detected in the isolates belonging to *Lb*. *paralimentarius*, *Lb*. *plantarum*, and *L*. *pseudomesenteroides*, in agreement with previous findings on the same three species [59]. Regarding the sole EPS-producing *Lb*. *curvatus/graminis* isolate (FF7) assayed, it was found to carry the *eps*A gene. HePS production by the species *Lb*. *curvatus* was first demonstrated by Van der Meulen et al. [68] with a preliminary screening of 174 LAB for EPS production, followed by PCR detection of the EPS-related genes, including *eps*A. A subsequent study [71] showed the presence of glycosyltransferases-related genes in the strain *Lb*. *curvatus* MTW 1.624, which was used to improve the quality of gluten-free baked products. In the same years, Palomba et al. [36] detected both HePS- (*eps*B, *eps*D/E) and HoPS-related (*gtf*) genes in the sourdough-sourced strain *Lb*. *curvatus* 69B2.

Both the EPS-producing *L*. *citreum* isolates assayed in this study harbored *eps*D/E and *gtf*. Coda et al. [41] have recently analyzed the performance of a *L*. *citreum* strain (FDR241) during the propagation of a type I wheat sourdough, revealing the presence of at least 5 different dextran sucrases after transcriptional analysis of the genes involved in EPS synthesis.

In regards to *W*. *confusa*, whose members produced mucoid colonies on SSM agar, one isolate (PB325) was negative in all PCR amplifications, whereas the remaining nine isolates harbored at least one of the three genes (*eps*A, *eps*D/E and *gtf*), thus showing the possibility for this species for producing both HoPS and HePS. The isolates PB323, PB333 and PB333 were positive for all these three genes, whereas PB330, PB331 and PB332 carried only the *gtf* gene. As early as 2009, Malik et al. [72] analyzed the *gtf* gene of two *W*. *confusa* strains (MBF8-1 and MBF8-2), highlighting a high sequence similarity with the same gene in *Lactobacillus reuteri* and *Lb fermentum*. None of the 28 EPS-producing LAB screened in the present study were positive for the genes *eps*EFG, *eps*B or *lev* under the applied PCR conditions, thus not excluding the possibility of false negative results.

Finally, negative PCR results were obtained from the isolates *Lb*. *fermentum* Bz2, *L*. *pseudomesenteroides* PB288, *P*. *pentosaceus* FF95, FO27 and FO30 and *W*. *confusa* PB325. This latter evidence might be tentatively ascribed to a low gene stability leading to false negatives, as previously suggested [73].

As far as the BLAST analysis of the EPS gene sequences is concerned, the following considerations can be made. As a general trend, most sequences were closely related to deposited sequences of LAB, as in the case of the *gtf* gene sequence from the isolate *L*. *citreum* PB220 showing a 89% similarity with a deposited sequence of the glucansucrase gene from the strain *L*. *citreum* 2.8 (DQ873511) or the *eps*D/E gene sequence from the same isolate showing a 97% similarity with a deposited sequence of the putative glycosylphosphotransferase gene from the strain *Vagococcus lutrae* MIS7 (KC841159). In addition, the *eps*D/E gene sequence from the isolate *Lb*. *plantarum* PB181 showed a 96% similarity with the deposited sequences of the glycosyltransferase gene from the strains *Lb*. *curvatus* FS9 (KC841158) and *P*. *pentosaceus* CRL 777 (DQ873506), whereas the *eps*D/E gene sequence from the isolate *Leuconostoc pseudomesenteroides* PB170 showed a 88% similarity with the deposited sequence of the *eps*E gene from the strain *Streptococcus thermophilus* BN1 (FN396421.1). Finally, *eps*A amplicons from the isolate *Lb*. *curvatus/graminis* FF7 obtained using primers targeting the *eps*A gene of *S*. *thermophilus* [74] showed a 100% similarity with a deposited sequence of the *eps*A gene of *S*. *thermophilus* (AF053346).

### Molecular weight and monosaccharide composition of purified EPS

The monosaccharide composition and molecular weight of EPS greatly influence the functionality of these biopolymers when used as food ingredients [58, 75]. The results of the determination of the molecular weights and monosaccharide compositions of the EPS produced by the selected 13 LAB are reported in Table 4.

**Table 4:** Molecular weight and monosaccharide composition of the exopolysaccharides (EPS) produced by selected lactic acid bacteria (LAB) isolates.

Isolate code	Species	Monosaccharide composition (%)*			Molecular weight (Da)
		Glucose	Mannose	Galactose	
LD58	*Lactobacillus brevis*	26.6±1.3^ef^	5.7±1.7^cde^	67.7±3.0^ab^	1.3×10^4^
LD66	*Lactobacillus brevis*	31.9±1.6^ef^	7.7±3.1^c^	60.4±4.7^bc^	1.1×10^4^
Bz2	*Lactobacillus fermentum*	68.7±3.1^a^	23.1±1.1^a^	8.2±2.0^f^	5.7×10^4^
PB127	*Lactobacillus paralimentarius*	43.6±3.0^c^	17.4±0.7^b^	39.0±2.3^e^	4.4×10^4^
PB3	*Leuconostoc citreum*	53.0±2.3^b^	2.1±0.3^def^	44.9±2.0^de^	4.3×10^4^
PB170	*Leuconostoc pseudomesenteroides*	41.3±1.7^cd^	16.1±0.8^b^	42.6±0.8^de^	2.1×10^4^
FF71	*Pediococcus pentosaceus*	34.8±0.4^de^	6.7±1.4^cd^	58.5±1.8^bc^	9.5×10^4^
FF78	*Pediococcus pentosaceus*	24.5±1.3^f^	2.4±0.3^def^	73.1±1.0^a^	9.0×10^4^
FO27	*Pediococcus pentosaceus*	44.6±3.0^c^	3.7±1.7^def^	51.7±4.7^cd^	4.3×10^4^
FO30	*Pediococcus pentosaceus*	53.2±2.4^b^	0.0±0.0^f^	46.8±2.4^de^	1.7×10^4^
PB323	*Weissella confusa*	58.0±2.1^b^	1.6±0.3^ef^	40.4±2.4^e^	7.3×10^4^
PB333	*Weissella confusa*	54.5±2.0^b^	1.7±0.3^ef^	43.8±1.7^de^	7.3×10^4^
PB337	*Weissella confusa*	59.9±1.3^b^	0.9±0.1^ef^	39.2±1.4^e^	6.1×10^4^

* Mean value of two replicates ± standard deviation. Values in a column with different letters are significantly different (Tukey-Kramer’s Honest Significant Difference test, p < 0.05).

The molecular weights of the EPS were between 1.1x10^4^ Da for those produced by *Lb*. *brevis* LD66 and 9.0x10^4^ Da for those produced by *P*. *pentosaceus* FF78. These values match with the molecular weight of the HePS, which are generally between 10^4^ to 10^6^ Da [76]. EPS with a molecular weight ≤ 10^4^ Da are considered as low molecular weight EPS [14]. Wang et al. [77] have previously reported a positive correlation between the low molecular weight of EPS and their antioxidant activity. By contrast, high molecular weight EPS are known to have a stronger antitumor activity with respect to the low molecular weight polymers [78].

The monosaccharide composition analysis revealed that these EPS were almost stably composed of glucose, mannose, and galactose, except for those produced by *P*. *pentosaceus* FO30, which did not include mannose. The overall results collected indicated that all the LAB assayed were HePS producers.

The EPS produced by both the *Lb*. *brevis* isolates assayed in the present study were characterized by a prevalence of galactose (60.4%-67.7%), followed by glucose (26.6%-31.9%) and mannose (5.7%-7.7%). A different EPS composition was found by Dertli et al. [79] in the EPS produced by the sourdough-sourced strain *Lb*. *brevis* E25, with glucose as the sole detected monosaccharide.

Similar to what was found for *Lb*. *brevis*, even the isolates ascribed to *W*. *confusa* showed a high homogeneity in the monosaccharide composition, with a prevalence of glucose (54.5%-59.9%), followed by galactose (39.2%-43.8%) and mannose (0.9%-1.7%). Malang et al. [80] have previously reported the production of HePS by the strains *W*. *confusa* 8CS-2, 11GU-1 and 11GT-2 isolated from sour milk. However, *W*. *confusa* is generally recognized as a HoPS-producing species, with the ability to release large amounts of high molecular weight dextrans. The latter biopolymers are exploitable by the baking industry for the improvement of the quality and texture of breads, including gluten-free breads [81]. A larger variability in the EPS monosaccharide composition was seen among the isolates belonging to *P*. *pentosaceus*. This finding agrees well with those reported by Imran et al. [82] on the high variability in the monosaccharide composition of the EPS produced by different *P*. *pentosaceus* strains. In the present study, the EPS produced by the isolates FF78 and FO30 showed the highest content in galactose (73.1%) and glucose (53.2%), respectively. In addition, three out of the four *P*. *pentosaceus* isolates assayed produced EPS containing from 2.4% (isolate FF78) to 6.7% (isolate FF71) mannose. Again, these results agreed with those of a research study [70] carried out on the EPS extracted from fruit-sourced isolates ascribed to *P*. *pentosaceus*, which were mainly composed of glucose, galactose, and mannose. In the present study, the isolate *Lb*. *fermentum* Bz2 produced EPS with the highest amount of mannose (23.1%) when compared with all the other tested isolates, followed by *Lb*. *paralimentarius* PB127 and *L*. *pseudomesenteroides* PB170, whose EPS included 17.4 and 16.1% mannose, respectively. Mannose is a minor component of the EPSs produced by LAB, whereas in those produced by yeasts, it represents a major component. To date, different authors have reported the occurrence of a positive correlation between the high mannose content of EPS and their anticancer activity [83].

The EPS produced by the isolate *Lb*. *fermentum* Bz2 were also characterized by the highest glucose content (68.7%) when compared to the EPS produced by all the other LAB assayed, and by the lowest amount of galactose (8.2%). Interestingly, a similar monosaccharide composition has recently been reported for a *Lb*. *fermentum* strain (YL-11) isolated from fermented milk [84].

In addition to mannose, the EPS produced by the isolate *L*. *pseudomesenteroides* PB170 also included glucose (41.3%) and galactose (42.6%). Similarly, the EPS purified from the isolate *L*. *citreum* PB3 were also characterized by glucose (41.3%), galactose (44.9%) and mannose (2.1%), but at a different relative ratio. As reported above, both these *Leuconostoc* isolates carried HePS-related genes, with the isolate ascribed to *L*. *citreum* also being positive for the *gtf* gene involved in HoPS production. To date, very few studies have described the ability of Leuconostocs to produce HePS. According to Ziadi et al. [85], members of the genus *Leuconostoc* produce HePS that are mainly composed of glucose and mannose and, to a lower extent, of rhamnose and arabinose. This was not expected since Leuconostocs are acknowledged as primary producers of dextran as well as other HoPS, such as alternan and levan [85, 86].

### Impact of LAB fermentation on the β-glucan content of a barley flour medium

The production of EPS is frequently associated with LAB metabolism, though the synthesis of a specific group of HoPS, referred to as β-glucans, is much less frequently associated with these microorganisms. To date, only a few LAB strains, ascribed to the species *Pediococcus parvulus*, *Oenococcus oeni*, and *Lactobacillus diolivorans*, have been reported to produce β-glucan in wine and cider, being responsible for the undesired ropiness of these alcoholic beverages [87]. However, β-glucans are principally known for their health-promoting properties, such as the ability of significantly reducing serum cholesterol levels and in turn, heart disease risk. Furthermore, β-glucans have immunomodulatory, anti-osteoporotic, anticytotoxic, antitumorigenic and antimutagenic effects, in addition to prebiotic traits that are associated with their ability to improve the growth, metabolism and beneficial activities of probiotics [12]. The β-glucans produced by LAB have already been used to produce fermented cereal-based products and yogurt to increase the functional and technological characteristics of these food products [88, 89].

In the present study, out of the 28 EPS-producing isolates assayed, 11 LAB belonging to *L*. *citreum* (isolates PB3 and PB220) and *W*. *confusa* (isolates PB315, PB323, PB324, PB327, PB330, PB331, PB332, PB333 and PB337) carried the *gtf* gene, which is involved in β-glucan production. Therefore, these isolates were further subjected to the evaluation of their effect on the β-glucan content of an *ad hoc* formulated BF medium. Our results revealed that 6 out of the 11 LAB individually inoculated into the BF medium were able to significantly increase the concentration of β-glucan after 24 h of fermentation. Among these, *W*. *confusa* PB332 and *L*. *citreum* PB220 performed the best, leading to a β-glucan increase as high as 7.32% and 6.86%, respectively, when compared with the non-inoculated controls (Table 3).

## Conclusions

Overall, the screening of the 152 LAB isolated from cereal-based substrates revealed a widespread capacity of these isolates (95%) for degrading phytic acid, though a very high variability was seen among the isolates, even within the same species. *Lb*. *brevis* LD65 and *Lb*. *plantarum* PB241 showed the highest phytase activity, while the isolates assigned to *W*. *confusa* could be distinguished for their low or no phytase activity. EPS production was limited to 18% of the tested LAB, with most of them carrying one or more genes involved in the production of HePS (*eps*D/E and/or *eps*A) and *L*. *citreum* and *W*. *confusa* isolates carrying also the genes involved in the production of HoPS. The monosaccharide composition analysis revealed that all the tested isolates were HePS producers, with glucose, mannose, and galactose being the most abundant monomers of the latter EPS. Interestingly, a few *W*. *confusa* and *L*. *citreum* isolates carrying the *gtf* gene were able to produce valuable amounts of β-glucans. Based on the overall results collected, a selected pool of isolates (including *Lb*. *brevis* LD66 and *L*. *citreum* PB220) showing both a high phytase activity and an appreciable EPS production, was identified for the formulation of sourdough starters with a potential in the manufacturing of breads with improved digestibility and mineral availability and/or improved softness and sensory traits. In addition, a few cultures with a potential for the manufacturing of fiber-rich functional bread, with an increased β-glucan content, were also found.

Further research efforts are needed to test the performance of these selected LAB in doughs prepared with both refined and phytate-rich flours from different cereal grains (e.g., wheat, emmer, and barley) for an *in vivo* assessment of their technological and functional potentials. Similarly, the finding of the capacity of several LAB isolates for enhancing β-glucan content in a barley flour medium is worth further investigation under *in vivo* conditions.

## Supporting information

S1 Table Complete list of the lactic acid bacteria (LAB) isolates and results of their screening for the phytase activity and the exopolysaccharides (EPS) production. ^**a**^The isolates were identified by comparing their 16S rRNA gene sequences with those deposited in the GenBank DNA database (http://www.ncbi.nlm.nih.gov/) using the Basic Local Alignment Search Tool (BLAST); * halo diameters (mm); ** R, ropy colonies (in brackets: the lengths, in mm, of the filaments picked from the colonies); M, mucoid colonies; -, no EPS production on SSM agar plates added with sucrose (5%, w v^-1^).
(DOCX)
