# Supplementary material for: Selection of cereal-sourced lactic acid bacteria as candidate starters for the baking industry
Source: PLoS One. 2020 Jul 23;15(7):e0236190. doi: 10.1371/journal.pone.0236190 (PMC7377444; doi:10.1371/journal.pone.0236190)
Supplement: S1 Table — aThe isolates were identified by comparing their 16S rRNA gene sequences with those deposited in the GenBank DNA database (http://www.ncbi.nlm.nih.gov/) using the Basic Local Alignment Search Tool (BLAST); * halo diameters (mm); ** R, ropy colonies (in brackets: the lengths, in mm, of the filaments picked from the colonies); M, mucoid colonies; -, no EPS production on SSM agar plates added with sucrose (5%, w v-1). (DOCX) [file pone.0236190.s001.docx]

**S1 Table.** Complete list of the lactic acid bacteria (LAB) isolates and results of their screening for the phytase activity and the exopolysaccharides (EPS) production.

| **Isolate code** | **Species^a^** | **Source** | **Phytase activity*** | **EPS**** |
| --- | --- | --- | --- | --- |
| Bz2 | *Lactobacillus fermentum* | Boza | 15 | R (8) |
| Bz5 | *Lactobacillus fermentum* | Boza | 24 | R (5) |
| Bz10 | *Lactobacillus paralimentarius* | Boza | 9 | - |
| Bz26 | *Lactobacillus parabuchneri* | Boza | 18 | - |
| Bz28 | *Lactobacillus parabuchneri* | Boza | 12 | - |
| Bz31 | *Lactobacillus parabuchneri* | Boza | 15 | - |
| Bz32 | *Lactobacillus buchneri* | Boza | 24 | - |
| Bz33 | *Lactobacillus casei/paracasei* | Boza | 12 | - |
| Bz34 | *Lactobacillus casei/paracasei* | Boza | 11 | - |
| Bz35 | *Lactobacillus casei/paracasei* | Boza | 8 | - |
| Bz36 | *Lactobacillus parabuchneri* | Boza | 17 | - |
| Bz37 | *Lactobacillus parabuchneri* | Boza | 14 | - |
| Bz38 | *Lactobacillus parabuchneri* | Boza | 20 | - |
| Bz39 | *Pediococcus parvulus* | Boza | 9 | - |
| Bz44 | *Lactobacillus coryniformis* | Boza | 7 | - |
| FF2 | *Lactobacillus curvatus/graminis* | emmer flour | 9 | - |
| FF3 | *Lactobacillus curvatus* | emmer flour | 8 | - |
| FF5 | *Lactobacillus curvatus* | emmer flour | 19 | - |
| FF7 | *Lactobacillus curvatus/graminis* | emmer flour | 17 | R (5) |
| FF15 | *Lactobacillus coryniformis* | emmer flour | 11 | - |
| FF33 | *Lactobacillus curvatus* | emmer flour | 21 | - |
| FF41 | *Enterococcus durans* | emmer flour | 8 | - |
| FF42 | *Enterococcus durans* | emmer flour | 8 | - |
| FF43 | *Lactobacillus curvatus* | emmer flour | 21 | - |
| FF44 | *Lactobacillus curvatus* | emmer flour | 13 | - |
| FF45 | *Enterococcus durans* | emmer flour | 12 | - |
| FF46 | *Enterococcus durans* | emmer flour | 17 | - |
| FF48 | *Lactobacillus curvatus* | emmer flour | 20 | - |
| FF49 | *Enterococcus durans* | emmer flour | 8 | - |
| FF50 | *Enterococcus durans* | emmer flour | 8 | - |
| FF51 | *Lactobacillus curvatus/graminis* | emmer flour | 6 | - |
| FF52 | *Lactobacillus curvatus/graminis* | emmer flour | 15 | - |
| FF53 | *Lactobacillus curvatus/graminis* | emmer flour | 13 | - |
| FF54 | *Enterococcus durans* | emmer flour | 7 | - |
| FF71 | *Pediococcus pentosaceus* | emmer flour | 12 | R (8) |
| FF78 | *Pediococcus pentosaceus* | emmer flour | 10 | R (7) |
| FF86 | *Pediococcus pentosaceus* | emmer flour | 21 | - |
| FF95 | *Pediococcus pentosaceus* | emmer flour | 14 | R (5) |
| FG2 | *Enterococcus casseliflavus* | wheat flour | 16 | - |
| FO2 | *Lactobacillus plantarum* | barley flour | 22 | - |
| FO8 | *Pediococcus pentosaceus* | barley flour | 13 | R (5) |
| FO13 | *Lactobacillus plantarum* | barley flour | 20 | - |
| FO27 | *Pediococcus pentosaceus* | barley flour | 17 | R (12) |
| FO30 | *Pediococcus pentosaceus* | barley flour | 15 | R (7) |
| FO40 | *Pediococcus pentosaceus* | barley flour | 21 | - |
| FO41 | *Pediococcus pentosaceus* | barley flour | 14 | - |
| LD58 | *Lactobacillus brevis* | sourdough | 15 | R (8) |
| LD65 | *Lactobacillus brevis* | sourdough | 27 | R (4) |
| LD66 | *Lactobacillus brevis* | sourdough | 22 | R (10) |
| LM1 | *Lactobacillus paralimentarius* | sourdough | 7 | - |
| LM2 | *Lactobacillus paralimentarius* | sourdough | 8 | - |
| LM3 | *Lactobacillus paralimentarius* | sourdough | 9 | - |
| LM4 | *Lactobacillus paralimentarius* | sourdough | 8 | - |
| LM5 | *Lactobacillus paralimentarius* | sourdough | 12 | - |
| LM6 | *Lactobacillus paralimentarius* | sourdough | 8 | - |
| LM7 | *Lactobacillus paralimentarius* | sourdough | 8 | - |
| LM8 | *Lactobacillus paralimentarius* | sourdough | 8 | - |
| LM9 | *Lactobacillus brevis* | sourdough | 11 | - |
| LM10 | *Lactobacillus paralimentarius* | sourdough | 8 | - |
| PB1 | *Lactobacillus curvatus* | sourdough | 10 | - |
| PB3 | *Leuconostoc citreum* | sourdough | 10 | M |
| PB11 | *Lactobacillus plantarum* | sourdough | 21 | - |
| PB14 | *Lactobacillus plantarum* | sourdough | 14 | - |
| PB15 | *Lactobacillus plantarum* | sourdough | 22 | - |
| PB22 | *Lactobacillus plantarum* | sourdough | 15 | - |
| PB24 | *Lactobacillus plantarum* | sourdough | 18 | - |
| PB46 | *Lactobacillus plantarum* | sourdough | 16 | - |
| PB57 | *Lactobacillus brevis* | sourdough | 19 | - |
| PB84 | *Lactobacillus plantarum* | sourdough | 17 | - |
| PB85 | *Lactobacillus plantarum* | sourdough | 19 | - |
| PB86 | *Lactobacillus plantarum* | sourdough | 15 | - |
| PB94 | *Lactobacillus paralimentarius* | sourdough | 12 | - |
| PB97 | *Lactobacillus plantarum* | sourdough | 23 | - |
| PB98 | *Lactobacillus plantarum* | sourdough | 20 | - |
| PB104 | *Lactobacillus plantarum* | sourdough | 12 | - |
| PB105 | *Lactobacillus plantarum* | sourdough | 13 | - |
| PB115 | *Lactobacillus curvatus* | sourdough | 10 | - |
| PB124 | *Lactobacillus plantarum* | sourdough | 15 | - |
| PB125 | *Lactobacillus paralimentarius* | sourdough | 17 | - |
| PB126 | *Lactobacillus paralimentarius* | sourdough | 15 | - |
| PB127 | *Lactobacillus paralimentarius* | sourdough | 11 | M |
| PB128 | *Lactobacillus plantarum* | sourdough | 10 | - |
| PB134 | *Lactobacillus plantarum* | sourdough | 14 | - |
| PB151 | *Lactobacillus plantarum* | sourdough | 17 | - |
| PB152 | *Weissella confusa* | sourdough | 12 | - |
| PB160 | *Lactobacillus fermentum* | sourdough | 18 | - |
| PB161 | *Lactobacillus plantarum* | sourdough | 8 | - |
| PB162 | *Lactobacillus fermentum* | sourdough | 14 | - |
| PB170 | *Leuconostoc pseudomesenteroides* | sourdough | 10 | M |
| PB173 | *Lactobacillus plantarum* | sourdough | 22 | - |
| PB181 | *Lactobacillus plantarum* | sourdough | 15 | M |
| PB190 | *\|Lactobacillus pentosus* | sourdough | 17 | - |
| PB191 | *Lactobacillus plantarum* | sourdough | 18 | - |
| PB193 | *Lactobacillus plantarum* | sourdough | 15 | - |
| PB199 | *Lactobacillus plantarum* | sourdough | 16 | - |
| PB200 | *Lactobacillus plantarum* | sourdough | 14 | - |
| PB202 | *Lactobacillus plantarum* | sourdough | 16 | - |
| PB209 | *Lactobacillus plantarum* | sourdough | 13 | - |
| PB210 | *Lactobacillus plantarum* | sourdough | 15 | - |
| PB213 | *Lactobacillus sanfranciscensis* | sourdough | - | - |
| PB219 | *Lactobacillus sanfranciscensis* | sourdough | 21 | - |
| PB220 | *Leuconostoc citreum* | sourdough | 16 | M |
| PB221 | *Lactobacillus sanfranciscensis* | sourdough | 16 | - |
| PB223 | *Lactobacillus sanfranciscensis* | sourdough | 16 | - |
| PB229 | *Lactobacillus paralimentarius* | sourdough | 9 | - |
| PB230 | *Lactobacillus paralimentarius* | sourdough | 11 | - |
| PB231 | *Lactobacillus paralimentarius* | sourdough | 13 | - |
| PB232 | *Lactobacillus paralimentarius* | sourdough | 20 | - |
| PB233 | *Lactobacillus paralimentarius* | sourdough | 15 | - |
| PB239 | *Lactobacillus plantarum* | sourdough | 13 | - |
| PB240 | *Lactobacillus plantarum* | sourdough | 16 | - |
| PB241 | *Lactobacillus plantarum* | sourdough | 27 | - |
| PB242 | *Lactobacillus plantarum* | sourdough | 16 | - |
| PB255 | *Lactobacillus plantarum* | sourdough | 13 | - |
| PB256 | *Lactobacillus plantarum* | sourdough | 16 | - |
| PB257 | *Lactobacillus plantarum* | sourdough | 18 | - |
| PB265 | *Lactobacillus paralimentarius* | sourdough | 7 | - |
| PB268 | *Lactobacillus plantarum* | sourdough | 22 | - |
| PB275 | *Lactobacillus plantarum* | sourdough | 19 | - |
| PB276 | *Lactobacillus sanfranciscensis* | sourdough | 14 | - |
| PB277 | *Lactobacillus plantarum* | sourdough | 18 | - |
| PB278 | *Lactobacillus plantarum* | sourdough | 21 | - |
| PB287 | *Lactobacillus plantarum* | sourdough | 12 | - |
| PB288 | *Leuconostoc pseudomesenteroides* | sourdough | - | 5 |
| PB294 | *Lactobacillus casei/paracasei* | sourdough | 13 | - |
| PB295 | *Leuconostoc pseudomesenteroides* | sourdough | 11 | - |
| PB296 | *Lactobacillus plantarum* | sourdough | 16 | - |
| PB297 | *Lactobacillus plantarum* | sourdough | 16 | - |
| PB298 | *Lactobacillus plantarum* | sourdough | 16 | - |
| PB304 | *Lactobacillus plantarum* | sourdough | 16 | - |
| PB305 | *Lactobacillus plantarum* | sourdough | 22 | - |
| PB306 | *Lactobacillus plantarum* | sourdough | 19 | - |
| PB307 | *Lactobacillus plantarum* | sourdough | 18 | - |
| PB308 | *Lactobacillus plantarum* | sourdough | 21 | - |
| PB309 | *Weissella confusa* | sourdough | 15 | - |
| PB311 | *Weissella confusa* | sourdough | 13 | - |
| PB313 | *Weissella confusa* | sourdough | 4 | - |
| PB314 | *Weissella confusa* | sourdough | - | - |
| PB315 | *Weissella confusa* | sourdough | - | M |
| PB319 | *Weissella confusa* | sourdough | - | - |
| PB321 | *Weissella confusa* | sourdough | 7 | - |
| PB323 | *Weissella confusa* | sourdough | 6 | M |
| PB324 | *Weissella confusa* | sourdough | 6 | M |
| PB325 | *Weissella confusa* | sourdough | - | M |
| PB327 | *Weissella confusa* | sourdough | 7 | M |
| PB330 | *Weissella confusa* | sourdough | 5 | M |
| PB331 | *Weissella confusa* | sourdough | 5 | M |
| PB332 | *Weissella confusa* | sourdough | - | M |
| PB333 | *Weissella confusa* | sourdough | - | M |
| PB334 | *Weissella confusa* | sourdough | 5 | - |
| PB337 | *Weissella kimchii* | sourdough | 15 | M |
| PB338 | *Weissella confusa* | sourdough | - | - |

**^a^**The isolates were identified by comparing their 16S rRNA gene sequences with those deposited in the GenBank DNA database (http://www.ncbi.nlm.nih.gov/) using the Basic Local Alignment Search Tool (BLAST); * halo diameters (mm); ** R, ropy colonies (in brackets: the lengths, in mm, of the filaments picked from the colonies); M, mucoid colonies; -, no EPS production on SSM agar plates added with sucrose (5%, w v^-1^)
